# The Views of Hospital Secretaries

**Published:** 1911-07-29

**Authors:** Richard J. Coles

**Affiliations:** Secretary-Superintendent of Addenbrooke's Hospital, Cambridge


### THE VIEWS OF HOSPITAL SECRETARIES.

### Mr. RICHARD J. COLES, Secretary-Superintendent of Addenbrooke's Hospital, Cambridge.

I have now carefully considered the various phases of the proposed National Insurance Bill, and speaking primarily as one interested in voluntary hospitals there is little doubt in my mind as to what the effect will be in the event of the Bill becoming law—so far as our voluntary hospitals in the United Kingdom are concerned. The income of every one of these voluntary hospitals, whether general or special, will be sadly diminished; and, while this diminution of income is going on, is the work of these institutions likely to be diminished in the same proportion or at all? Certainly not; the work will be considerably increased both as regards in-patients and out-patients.

It is true that the Bill provides for medical attendance for the wage-earners, but there is no such provision made for the wives and children. In these latter cases, where they are unable to obtain medical and surgical treatment in the usual way, they must and will still present themselves at our voluntary hospitals; then there will be those who, although they are insured under the said Bill, will be compelled to come to the voluntary hospitals for such special and operative treatment for which the Bill does not provide or even pretend to provide.

In addition to the foregoing conditions there are certain very prominent conditions which are, and have been for many years, responsible for sending so many members of Friendly Societies and sick benefit clubs to our voluntary general and special hospitals —at least those in the provinces with which I am specially familiar. At the same time I do not doubt for one moment but that the same conditions exist in London, and in like manner are responsible for sending large numbers of patients to the London hospitals.

Now if without such a Bill these conditions exist and are responsible for sending patients in shoals to our voluntary general and special hospitals, is it reasonable to suppose that the passing into law of such a Bill as the one now before Parliament is likely to obliterate these conditions referred to or even to minimise them? Decidedly not—exactly the opposite effect must of necessity ultimately be the result, and as a consequence the number of in-patients and out-patients at the voluntary hospitals will be increased to an alarming extent.

It may be difficult to predict the extent to which the passing into law of such a measure as the said National Insurance Bill is likely to reduce the income of our voluntary hospitals, but such is not the case in predicting the extent to which the number of patients is likely to be increased—without doubt the work will be increased by at least 10 to 15 per cent.

I notice in the report of a meeting of the Committee of King Edward's Hospital Fund for London, which appeared in The Hospital last week, it stated that power under Clause 17 of the proposed National Insurance Bill for Friendly Societies to subscribe to hospitals is not likely to operate beneficially, as such contributions are optional, and if compulsory such might tend to intervention in the management of such hospitals. Both these apprehensions are in my opinion complete nightmares, as in the first instance Friendly Societies have subscribed to the funds of our voluntary hospitals for years, therefore they have not only had the power, but have actually exercised that power all these years. Secondly, such Friendly Societies can only enjoy the same privileges as the said voluntary hospitals bestow on their subscribers in general, therefore optional power to subscribe already exists, and compulsory power is not only unnecessary but cannot possibly tend to intervention of management.

It is to be deplored that at various meetings of the Special Committees of the British Hospitals Association and the Incorporated Association of Hospital Officers suggestions should have been advanced to urge that provision should be made in the Bill for payment to hospitals in respect of all insured persons receiving treatment whether as in- or out-patients.

If provision is made for the sick allowance of 10s. per week to be paid to the hospital during the time the insured is confined to such hospital, this sum would not anything like reimburse the hospital, seeing that the average cost of maintaining a patient in most of the provincial hospitals is about 33s. 4d. per week. Again, if provision as above-mentioned is made in the Bill, and is accepted by the voluntary hospitals, is it unreasonable to expect that the hon. physicians and surgeons will then immediately demand payment for their services, and rightly so, for they could no longer be expected to render voluntary service to State-aided institutions?

State aid must be accompanied with State control, and it is quite patent that if State aid enters our voluntary hospital system—a system which has been not only admired but envied throughout the whole world—charity will, without doubt, vanish, and with what result?—to the disadvantage of all those of our less fortunate brothers and sisters for whose special benefit these voluntary hospitals have not only been founded but maintained all down the ages.

It is almost impossible to conceive, and it is to be deplored, that any statesman should have brought forward a scheme or Bill of such far-reaching effect without first consulting the members of the medical profession, who have rendered such valuable and unselfish service to the sick poor, and the governors and officers of all the voluntary hospitals in the United Kingdom; and it behoves us to leave no stone unturned with the object of frustrating this designed attempt to wreck one of the most noble of institutions in our land.

The thin edge of the wedge is being forged—let us one and all see to it that the wedge finds no resting place.

It is essential that no time should be lost on the part of hospital committees and officers alike to not only assert but to pursue that which we believe to be the right policy on this occasion—namely, to maintain wholly and unimpaired the voluntary hospital system in this country.—I am, dear Sir, yours very faithfully, R. J. Coles.

